# Thioredoxin Reductase-Type Ferredoxin: NADP^+^ Oxidoreductase of *Rhodopseudomonas palustris*: Potentiometric Characteristics and Reactions with Nonphysiological Oxidants

**DOI:** 10.3390/antiox11051000

**Published:** 2022-05-19

**Authors:** Mindaugas Lesanavičius, Daisuke Seo, Narimantas Čėnas

**Affiliations:** 1 Department of Xenobiotics Biochemistry, Institute of Biochemistry of Vilnius University, Saulėtekio 7, LT-10257 Vilnius, Lithuania; mindaugas.lesanavicius@gmc.vu.lt; 2 Division of Material Sciences, Graduate School of Natural Science and Technology, Kanazawa University, Kakuma, Kanazawa 920-1192, Japan; dseo@se.kanazawa-u.ac.jp

**Keywords:** *Rhodopseudomonas palustris*, ferredoxin:NADP^+^ oxidoreductase, thioredoxin reductase, quinones, nitroaromatic compounds, reduction mechanism, redox potential

## Abstract

*Rhodopseudomonas palustris* ferredoxin:NADP^+^ oxidoreductase (*Rp*FNR) belongs to a novel group of thioredoxin reductase-type FNRs with partly characterized redox properties. Based on the reactions of *Rp*FNR with the 3-acetylpyridine adenine dinucleotide phosphate redox couple, we estimated the two-electron reduction midpoint potential of the FAD cofactor to be −0.285 V. 5-Deaza-FMN-sensitized photoreduction revealed −0.017 V separation of the redox potentials between the first and second electron transfer events. We examined the mechanism of oxidation of *Rp*FNR by several different groups of nonphysiological electron acceptors. The *k*_cat_/*K*_m_ values of quinones and aromatic *N*-oxides toward *Rp*FNR increase with their single-electron reduction midpoint potential. The lower reactivity, mirroring their lower electron self-exchange rate, is also seen to have a similar trend for nitroaromatic compounds. A mixed single- and two-electron reduction was characteristic of quinones, with single-electron reduction accounting for 54% of the electron flux, whereas nitroaromatics were reduced exclusively via single-electron reduction. It is highly possible that the FADH· to FAD oxidation reaction is the rate-limiting step during the reoxidation of reduced FAD. The calculated electron transfer distances in the reaction with quinones and nitroaromatics were close to those of *Anabaena* and *Plasmodium falciparum* FNRs, thus demonstrating their similar “intrinsic” reactivity.

## 1. Introduction

Ferredoxin:NADP^+^ oxidoreductases (FNRs) transfer redox equivalents between NADP(H) and the low-redox-potential FeS protein ferredoxin (Fd), or flavodoxin, a low-molecular-weight flavin mononucleotide (FMN)–containing protein [1,2,3,4,5,6,7]. FNRs comprise separate flavin adenine dinucleotide (FAD)- and NADP(H)-binding domains. The stabilization of the neutral (blue) FAD semiquinone (FADH·) as the reaction intermediate takes place by transforming the two-electron (hydride) transfer into two single-electron transfer events [1,2,8]. The complex formation between FNR and Fd is frequently attributed to the electrostatic and hydrophobic interactions [2,6,9].

**Figure 1:**
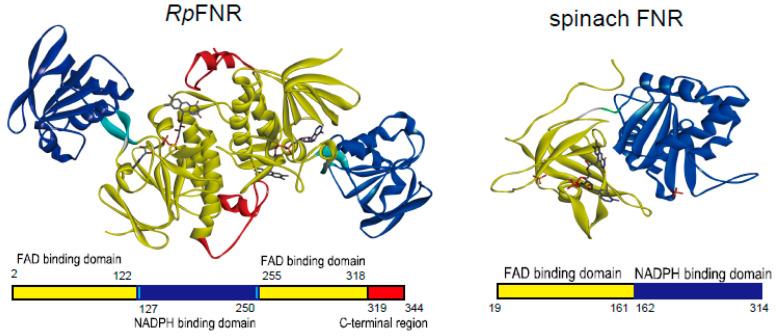
Ribbon diagrams of *Rp*FNR (homo-dimer, PDB ID: 5YGQ) and spinach FNR (PDB ID: 1FNB). The domain organizations are indicated at the bottom. FAD-binding and NADP^+^/H-binding domains are colored yellow and blue, respectively, and the C-terminal region of *Rp*FNR is in red. Bound FAD co-factor is represented as a stick model. The figure was prepared using BIOVIA Discovery Studio Visualizer (Ver. 21.1, Dassault Systèms).

FNRs are found in a wide variety of organisms and are classified into several groups and subclasses, whose representatives differ in amino acid sequence, catalytic rate, specificity for NAD(P)(H), physiological functions, and direction of electron transfer [3,7,10]. Most recently, a distinctive subclass of thioredoxin reductase-type FNRs has been discovered, whose structure exhibits the low *M*_r_ thioredoxin reductase (TrxR) fold ([10], and references therein). This fold consists of two domains with Rossmann-like three-layer ββα sandwich folds that bind FAD and NADP(H). The typical representatives of this subclass are dimeric FNRs of the green sulfur bacterium *Chlorobaculum tepidum* [11,12], the heterotrophic Gram-positive bacterium *Bacillus subtilis* [13,14,15], and the photosynthetic purple nonsulfur bacterium *Rhodopseudomonas palustris* [15,16]. In these enzymes, the residues of the NADP(H)-binding domain are inserted between the two sections of the FAD-binding domain residues, and a hinge region connects the two domains (Figure 1). The rotation of the domains relative to each other may take place in catalysis, e.g., in *B. subtilis* FNR, NADP^+^ is bound ca. 15 Å away from the isoalloxazine ring of FAD, which is too distant for efficient hydride transfer [13]. The redox properties of Trx-type FNRs have been characterized partly, limited mostly by the studies of their reactions with NADP^+^/NADPH and Fds.

*R. palustris* TrxR-type FNR (RPA3954, *Rp*FNR, EC 1.18.1.2) consists of the FAD-binding domain (residues 2–122 and 255–344, including the flexible C-terminal region, residues 319–344) containing a specific Tyr328 residue covering the *re* side of the isoalloxazine ring, and the NADP(H)-binding domain (residues 127–250). The reduction of *Rp*FNR by NADPH and reoxidation by NADP^+^ proceeds in several phases, the fastest ones exceeding 500 s^−1^, and involves the formation of several intermediate charge-transfer complexes [16]. On the other hand, *Rp*FNR has low reactivity toward Fe_2_S_2_-type ferredoxin (RPA3956), whereas its reactivity toward Fe_4_S_4_-type Fds of *R. palustris* has not been reported [15].

In order to extend the understanding of the redox properties of *Rp*FNR, we investigated its reactions with nonphysiological electron acceptors with different structures and single-electron reduction potentials (*E*^1^_7_). It is worth noting that *R. palustris* is capable of metabolizing aromatic compounds formed during plant degradation, which may involve FNR/Fd and cytochrome P-450-dependent redox systems [17,18]. Besides, some FNRs, such as the malaria parasite *Plasmodium falciparum* FNR, are a potential target for redox active drug candidates, quinones and nitroaromatic compounds [19,20]. In order to quantitatively analyze the obtained results, the redox potentials of *Rp*FNR were also determined in this work.

## 2. Materials and Methods

### 2.1. Enzymes and Reagents

Recombinant *R. palustris* ferredoxin:NADP^+^ oxidoreductase was prepared as previously described [16]. Its concentration was determined spectrophotometrically according to ε_466_ = 10.8 mM^−1^ cm^−1^ [16]. 2,4,6-Trinitrotoluene (TNT) and 2,4,6-trinitrophenyl-*N*-methylnitramine (tetryl), synthesized as described [21], and 3-amino-1,2,4-benzotriazine-1,4-dioxide (tirapazamine) and its 7-methyl- and 7-fluoro- derivatives, synthesized according to [22], were a generous gift from Dr. Jonas Šarlauskas (Institute of Biochemistry, Vilnius). 5-(1-Aziridinyl)-2,4-dinitrobenzamide (CB-1954) synthesized as described in [23], was a generous gift from Dr. Vanda Miškinienė (Institute of Biochemistry, Vilnius). The above compounds were characterized by their melting points and their ^1^H-NMR, UV, and IR spectra. The purity of compounds determined using HPLC-MS (LCMS-2020, Shimadzu, Kyoto, Japan) was >98%. NADPH, 3-acetylpyridine adenine dinucleotide phosphate (AcPyP^+^), horse heart cytochrome *c*, superoxide dismutase, and other reagents were obtained from Sigma-Aldrich (St. Louis, MO, USA) and used as received.

### 2.2. Photoreduction of RpFNR

*Rp*FNR (16–17 µM) photoreduction was performed under anaerobic conditions in 0.02 M Hepes buffer, pH 7.0, using 5-deaza-FMN (0.125 µM) and EDTA (8 mM) as photosensitizers. Before protein introduction from a concentrated stock solution, the solution in a sealed spectrophotometer cuvette was flushed with O_2_-free argon for 60 min. Here and in subsequent experiments, a Cary60 UV-Vis (Agilent Technologies, Santa Clara, CA, USA) or a PerkinElmer Lambda 25 UV–VIS spectrophotometer (PerkinElmer, Waltham, MA, USA) was used. Subsequently, the cell was irradiated for short periods at 20 °C with a 100 W incandescent lamp (Osram) at a distance of 20 cm; the progress of the reaction was followed spectrophotometrically for 1–1.5 h. The maximal amount of neutral semiquinone (E-FADH·) formed under irradiation was assumed to be defined by the inflection point of the A_600_ vs. A_466_ plot. The FAD semiquinone concentration was calculated using ε_600_ = 5.0 mM^−1^cm^−1^ [24]. The separation between the two single-electron-transfer potentials (Δ*E*^1^_7_) was further calculated from the semiquinone formation constant *K*_s_ (Equations (1) and (2)):

[E-FADH]_max_/[E-FAD]_tot_ = *K*_s_^1/2^/(2 + *K*_s_^1/2^), (1)

Δ*E*^1^_7_ = *E*_7_(E-FAD/E-FADH) − *E*_7_(E-FADH / E-FADH^−^) = 0.059 V × log *K*_s_, (2)

where [E-FADH]_max_ is the maximal amount of formed semiquinone, *E*_7_(E-FAD/E-FADH) is the potential of oxidized/semiquinone couple, *E*_7_(E-FADH/E-FADH^−^) is the potential of semiquinone/reduced FAD couple, and [E-FAD]_tot_ is the total enzyme concentration [25].

### 2.3. Steady-State Kinetic Studies

The kinetic experiments were performed spectrophotometrically in 0.02 M Hepes + 1 mM EDTA buffer (pH 7.0), at 25 °C. The kinetic data were fitted to a parabolic expression in SigmaPlot (v. 11.0, SPSS Inc., Chicago, IL, USA) to yield the steady-state parameters of the reactions, catalytic constants (*k*_cat(app.)_), and bimolecular rate constants (*k*_cat_/*K*_m_)) of the oxidants under fixed concentrations of NADPH. They are equal to the reciprocal intercepts and slopes of Lineweaver–Burk plots, [E]/*v* vs. 1/[oxidant], respectively, where [E] is the enzyme concentration*,* and *v* is the reaction rate. *k*_cat_ represents the number of molecules of NADPH oxidized by a single active center of the enzyme per second at saturated concentrations of both substrates. Kinetic parameters of steady-state reactions according to a “ping-pong” mechanism were calculated according to Equation (3):

(3) $$\frac{v}{\left[E\right]}=\frac{k_{\mathrm{cat}\;}\left[S\right]\left[Q\right]}{K_{m\left(S\right)}\left[Q\right]+K_{m\left(Q\right)\;}\left[S\right]+\left[S\right]\left[Q\right]}\;,\;$$

where S stands for NADPH, and Q stands for the electron acceptor. The competitive inhibition constant (*K*_is_) of NADP^+^ (I) vs. NADPH was calculated according to Equation (4):

(4) $$\frac{v}{\left[E\right]}=\frac{k_{\mathrm{cat}\left(\mathrm{app}\right)\;}\left[S\right]}{K_{m\left(S\right)}\left(1+\frac{\left[I\right]}{K_{\mathrm{is}}}\right)+\left[S\right]}\;,\;\;$$

and the noncompetitive inhibition constant (*K*_ii_) of NADP^+^ vs. electron acceptor (Q) was calculated according to Equation (5):

(5) $$\frac{v}{\left[E\right]}=\frac{k_{\mathrm{cat}\left(\mathrm{app}\right)\;}\left[Q\right]}{(K_{m\left(Q\right)}+\left[Q\right])\left(1+\frac{\left[I\right]}{K_{\mathrm{ii}}}\right)}\;.\;\;$$

The rates of enzymatic NADPH oxidation in the presence of quinones, nitroaromatic compounds, or tirapazamine derivatives were determined using the value ∆ε_340_ = 6.2 mM^−1^ cm^−1^. The rates were corrected for the intrinsic NADPH-oxidase activity of *Rp*FNR, 0.12 s^−1^. In separate experiments, in which 50 µM cytochrome *c* was additionally added into the reaction mixture, its quinone- or nitroaromatic-mediated reduction was assessed using the value ∆ε_550_ = 20 mM^−1^cm^−1^. The ferricyanide reduction rate was measured using the value ∆ε_420_ = 1.03 mM^−1^cm^−1^. The rate of the *Rp*FNR-catalyzed reduction of AcPyP^+^ by NADPH was determined using the value ∆ε_363_ = 5.6 mM^−1^ cm^−1^ [26]. AcPyPH, the reduced form of AcPyP^+^, was prepared in situ by the reduction of AcPyP^+^ with 10 mM glucose-6-phosphate and 0.01 mg/mL glucose-6-phosphate dehydrogenase. AcPyPH concentration was determined according to ε_365_ = 7.8 mM^−1^cm^–1^ [26]. The statistical analysis was performed using Statistica (version 4.3, Statsoft, Toronto, ON, Canada).

### 2.4. Presteady-State Kinetic Studies

The rapid kinetic studies of *Rp*FNR were performed using a SX20 stopped-flow spectrophotometer (Applied Photophysics, Leatherhead, UK) under aerobic conditions. The enzyme reduction by NADPH and its reoxidation was monitored between 450 and 800 nm, as further described in the Results section. During turnover studies, *Rp*FNR in the first syringe (4.0 µM after mixing) was mixed with the contents of the second syringe (50 µM NADPH and 250 µM tetramethyl-1,4-benzoquinone after mixing). The control experiments were performed in the absence of quinone. The reoxidation kinetics were analyzed by the method of Chance [27] according to Equation (6), where *k*_ox_ is the apparent first-order rate constant of enzyme reoxidation, [NADPH]_0_ is the initial NADPH concentration, [E_red_]_max_ is the maximal concentration of the reduced enzyme formed during the turnover, and t_1/2(off)_ is the time interval between the formation of the half-maximal amount of E_red_ and its decay to the half-maximal value:

*k*_ox_ = [NADPH]_0_/([E_red_]_max_ × t_1/2(off)_). (6)

## 3. Results

### 3.1. Determination of Redox Potentials of RpFNR

**Figure 2:**
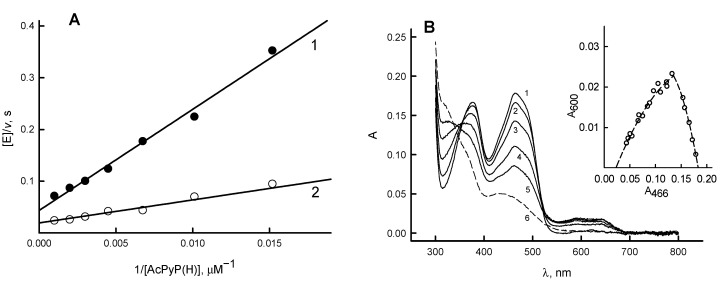
Determination of redox potentials of *Rp*FNR. (**A**) Rates of *Rp*FNR-catalyzed oxidation of AcPyPH with 1 mM ferricyanide (1), and of *Rp*FNR-catalyzed reduction of AcPyP^+^ with 200 µM NADPH (2). (**B**) Spectra obtained during the photoreduction of 16.6 µM *Rp*FNR at different times of illumination: immediately after mixing (1), after 10 min (2), 25 min (3), 40 min (4), 50 min (5), and after 70 min (6, fully reduced enzyme). Inset shows the interdependence of absorbance changes at 466 and 600 nm during photoreduction.

According to the best of our knowledge, the potentiometric characteristics of *Rp*FNR were unavailable so far. In order to determine the standard redox potential (*E*^0^_7_, potential of E-FAD/E-FADH^−^ redox couple) of *Rp*FNR, we examined its reactions with the analogue of NADP(H), 3-acetylpyridine adenine dinucleotide phosphate, AcPyP(H) (*E*^0^_7_ = −0.258 V). AcPyP^+^ was chosen instead of NADP^+^ because the reduction of NADP^+^ by *Rp*FNR under steady-state conditions is problematic due to the lack of a suitable electron donor [15]. During the enzymatic reduction of AcPyP^+^ by NADPH, the maximum reaction rate was reached at 200 µM NADPH. In this reaction, *k*_cat_ = 53.3 ± 3.1 s^−1^, and *k*_cat_/*K*_m_ for AcPyP^+^ is estimated to be 2.27 ± 0.35 × 10^6^ M^−1^s^−1^ (Figure 2A).

In the reverse reaction using AcPyPH generated in situ and 1.0 mM ferricyanide as an electron acceptor, *k*_cat_ = 18.6 ± 0.7 s^−1^, and *k*_cat_/*K*_m_ for AcPyPH is calculated to be 6.0 ± 0.6 × 10^5^ M^−1^s^−1^ on the two-electron basis (Figure 2A). According to the Haldane relationship, the equilibrium constant (*K*) of the redox reaction with the AcPyP^+^/AcPyPH couple corresponds to the ratio of *k*_cat_/*K*_m_ for AcPyPH and AcPyP^+^, respectively. According to the Nernst equation, the difference between the redox potentials of the reactants, Δ*E*^0^, equals 0.0295 V × log *K*. This provides the *K* value of 0.264 ± 0.067, and the *E*^0^_7_ value for the enzyme of −0.276 ± 0.003 V, respectively.

During the photoreduction of *Rp*FNR in the presence of 5-deaza-FMN and EDTA, the neutral FAD semiquinone (FADH·) with the characteristic absorbance at 550–650 nm is formed (Figure 2B). The amount of E-FADH· calculated using ε_600_ = 5.0 mM^−1^cm^−1^ [24] and the data from the inset of Figure 2B is 26.5%. According to Equations (1) and (2), this gives *K*_s_ = 0.520, and Δ*E*^1^_7_ = −0.017 V, which corresponds to *E*_7_ (E-FAD / E-FADH·) = −0.285 V and *E*_7_(E-FADH·/ E-FADH^−^) = −0.268 V. However, a slight correction for these potentials is not ruled out, as the ε_600_ of FADH of *Rp*FNR is not definitely determined.

### 3.2. Steady-State Kinetics and Oxidant Substrate Specificity Studies of RpFNR

**Figure 3:**
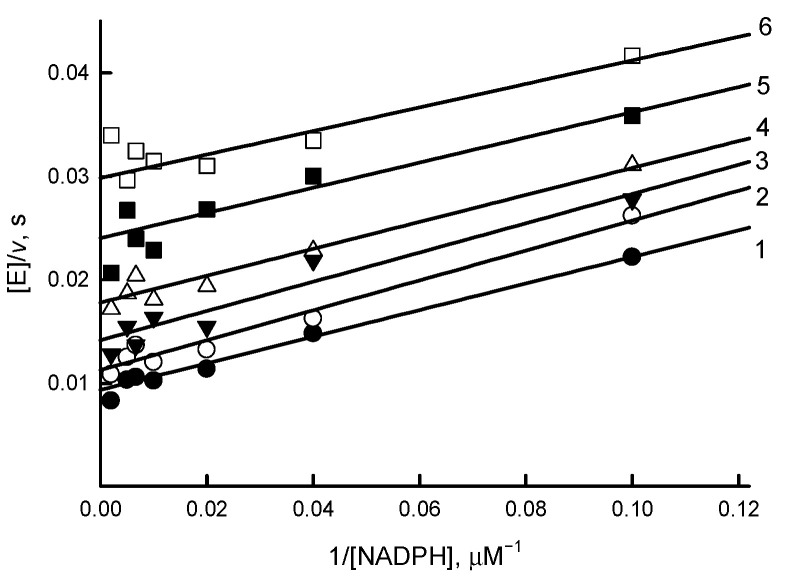
Lineweaver–Burk plot of the steady state kinetics of oxidation of NADPH catalyzed by *Rp*FNR in the presence of juglone. Juglone concentrations are 200 μM (1), 133 μM (2), 89 μM (3), 59 μM (4), 39 μM (5), and 26 μM (6).

The previous kinetic studies of *Rp*FNR were performed using the classical nonphysiological electron acceptor of FNRs, ferricyanide [16]. We preliminarily identified a representative of another group of compounds, juglone (5-hydroxy-1,4-naphthoquinone) as an efficient nonphysiological electron acceptor of *Rp*FNR. A series of parallel lines obtained in Lineweaver–Burk plots at varied concentrations of NADPH and fixed concentrations of juglone are indicative of a “ping-pong” mechanism for the quinone reductase activity of *Rp*FNR (Figure 3).

As deduced from Equation (3), the *k*_cat_ value for the juglone reduction at infinite NADPH concentration is equal to 157 ± 7.0 s^−1^, and the values of the bimolecular rate constants (*k*_cat_/*K*_m_) for NADPH and juglone are equal to 8.7 ± 0.7 × 10^6^ M^−1^s^−1^ and 1.62 ± 0.15 × 10^6^ M^−1^s^−1^, respectively. The value of *k*_cat_/*K*_m_ for NADPH is similar to that obtained previously, 5.5 × 10^6^ M^−1^s^−1^, using ferricyanide as the acceptor [16].

Next, we assessed the oxidant substrate specificity of *Rp*FNR, examining its reactions with quinones (Q), nitroaromatic compounds (ArNO_2_), and aromatic *N-*oxides (ArN→O), which comprise three distinct groups of electron acceptors characterized by single-electron reduction midpoint potentials (*E*^1^_7_) in the range of 0.09 to −0.494 V. These compounds were studied along with several single-electron acceptors such as ferricyanide, Fe(EDTA)^−^, and benzylviologen. The studied compounds included the explosives tetryl and 2,4,6-trinitrotoluene, antibacterial agents nitrofurantoin and nifuroxime, and anticancer agents CB-1954 and tirapazamine. The apparent maximal reduction rate constants, *k*_cat(app)_, of electron acceptors at 100 µM NADPH and their respective *k*_cat_/*K*_m_ are given in Table 1.

**Figure 4:**
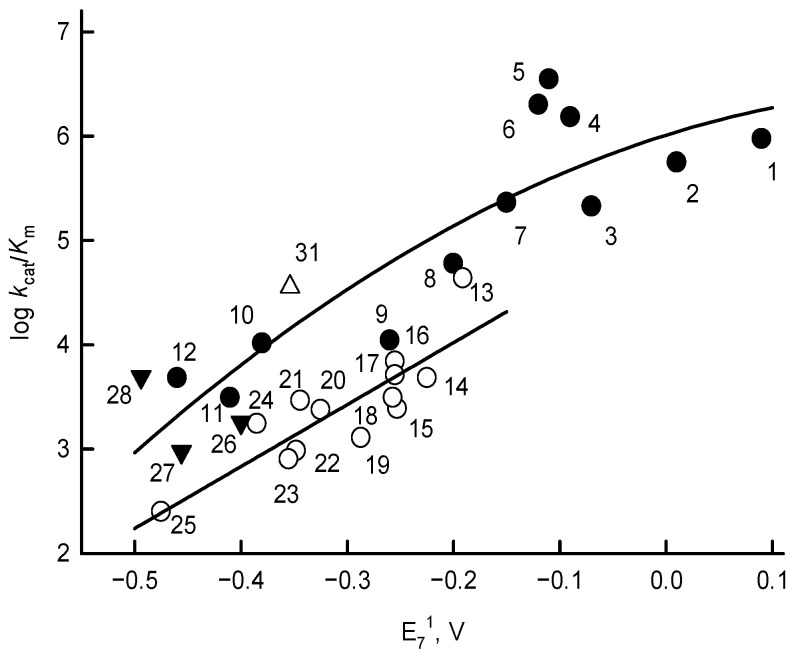
Dependence of the reactivity (log *k*_cat_/*K*_m_) of quinones (solid circles), nitroaromatic compounds (blank circles), aromatic *N*-oxides (solid triangles), and benzylviologen (blank triangle) on their single-electron reduction midpoint potentials (*E*^1^_7_). Numbers and reduction potentials of compounds are given in Table 1.

The log *k*_cat_/*K*_m_ values of nitroaromatics exhibit a linear although scattered dependence on their *E*^1^_7_ (Table 1 and Figure 4). In general, the log *k*_cat_/*K*_m_ values of quinones, including the single-electron acceptor benzylviologen, and ArN→O, are higher than those of ArNO_2_, and demonstrate a parabolic dependence on their *E*^1^_7_ values (Figure 4).

It is established that FNRs from spinach, *Anabaena* spp. and *Plasmodium falciparum* reduce quinones and nitroaromatics in a single-electron way [19,30,31]. For quinone reduction by NAD(P)H-oxidizing flavoenzymes, the single-electron flux is defined as a ratio of the rate of 1,4-benzoquinone-mediated reduction of the added cytochrome *c* to the doubled rate of 1,4-benzoquinone-mediated NAD(P)H enzymatic oxidation at pH < 7.2 [30]. This approach is based on the fast reduction of cytochrome *c* by 1,4-benzosemiquinone (k ~ 10^6^ M^−1^s^−1^), and its slow reduction by the hydroquinone form. We found that for enzymatic reduction of 50–100 µM 1,4-benzoquinone by 50–100 µM NADPH, the single-electron flux was equal to 54 ± 4.0% of the total flux. The assessment of the single-electron flux in the reduction of aromatic nitrocompounds can be based on the ArNO_2_^−^·-mediated reduction of added cytochrome *c.* We found that, in the presence of 50 μM NADPH and 100 μM TNT or *p*-nitrobenzaldehyde, the rate of *Rp*FNR-catalyzed reduction of added 50 μΜ cytochrome *c* was equal to 91 ± 2.0% and 97 ± 3.0% of the doubled NADPH oxidation rate, respectively. These reactions were inhibited by 100 U/mL superoxide dismutase by 49% and 37%, respectively, which reflects the rapid reoxidation of ArNO_2_^−^· with O_2_ and the participation of superoxide in the reduction of cytochrome *c.* Thus, one may conclude that *Rp*FNR reduces ArNO_2_ in a single-electron way.

**Figure 5:**
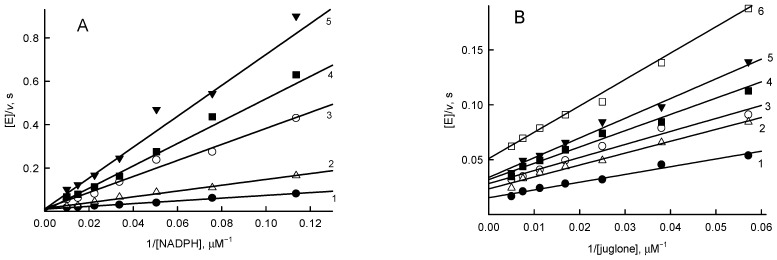
Inhibition of *Rp*FNR-catalyzed reactions by NADP^+^. (**A**) Competitive inhibition of the juglone reductase reaction of *Rp*FNR by NADP^+^ at varied concentration of NADPH and in the presence of 200 μM juglone. NADP^+^ concentrations are 0 (1), 0.25 mM (2), 0.5 mM (3), 0.75 mM (4), and 1.0 mM (5). (**B**) Noncompetitive inhibition at varied juglone concentration in the presence of 100 μM NADPH. NADP^+^ concentrations are 0 (1), 0.5 mM (2), 1.0 mM (3), 1.5 mM (4), 2.0 mM (5), and 3.0 mM (6).

Finally, we examined the inhibition of quinone reductase reaction of *Rp*FNR by the reaction product NADP^+^. At a fixed juglone concentration (200 μM), NADP^+^ acted as a competitive inhibitor toward NADPH (Figure 5A) with *K*_is_ = 150 ± 10 µM, as deduced from Equation (4). In turn, at a fixed concentration of 100 µM NADPH, NADP^+^ acts as an apparently noncompetitive inhibitor toward juglone (Figure 5B) with *K*_ii_ = 1.7 ± 0.1 mM, as obtained using Equation (5).

### 3.3. RpFNR Oxidation under Multiple Turnover Conditions

**Figure 6:**
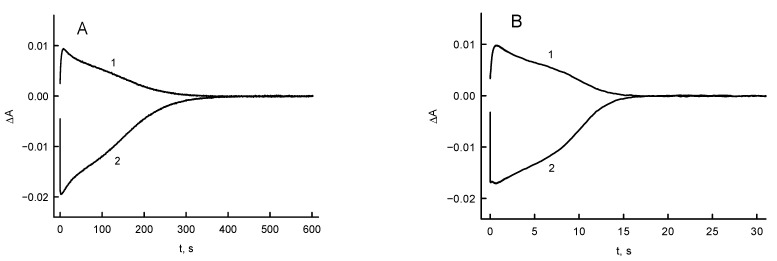
The absorbance changes at 600 nm (1) and 460 nm (2) during the reduction of *Rp*FNR (4.0 μM) by 50 μM NADPH and its subsequent reoxidation by oxygen (**A**) or 250 μM duroquinone (**B**). Concentrations are reported after mixing.

The spectral changes of *Rp*FNR-bound FAD during its multiple turnover under aerobic conditions in the presence of NADPH and tetramethyl-1,4-benzoquinone (duroquinone) provides insight into the reoxidation mechanism of the enzyme. Duroquinone does not possess absorbance at ≥460 nm; besides, its semiquinone form is rapidly reoxidized by oxygen [28]. Control experiments were performed without the addition of a quinone, and the initial fast phase of FAD reduction by NADPH observed at 460 nm was followed by a slow reoxidation by oxygen (Figure 6A). A transient increase in absorbance at 600 nm at the same time scale accompanies this process (Figure 6A). In the presence of quinone, the reoxidation of FADH^−^ and the disappearance of the 600 nm absorbing species are accelerated by more than one order of magnitude (Figure 6B).

The maximal ΔA_460_ after the enzyme mixing with NADPH (Figure 6A) corresponds to 90% of *Rp*FNR FAD absorbance decrease after the enzyme mixing with an excess NADPH under anaerobic conditions [15,16]. Assuming that the maximal concentration of the reduced enzyme form under aerobic conditions is 90% of total enzyme, for the reoxidation of *Rp*FNR with oxygen (Figure 6A), using Equation (6) we obtain a *k*_ox_ = 0.11 ± 0.01 s^−1^, which was close to the steady-state NADPH oxidase activity of *Rp*FNR. In the presence of 250 µM tetramethyl-1,4-benzoquinone, we obtain a *k*_ox_ = 2.05 ± 0.07 s^−1^, which is close to the steady-state reduction rate of this oxidant (Table 1).

**Figure 7:**
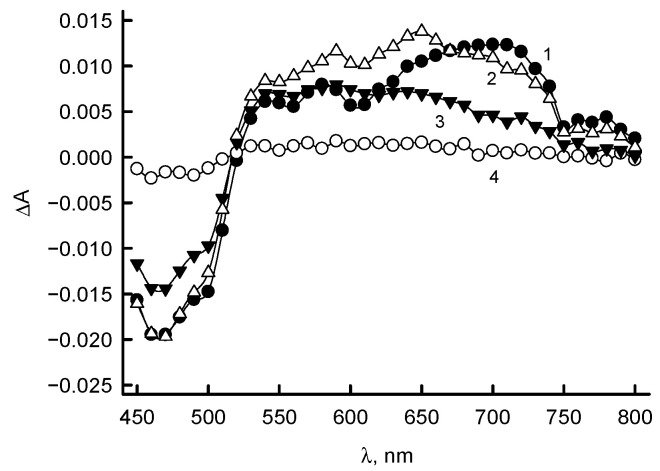
Spectra of reaction intermediates formed during the turnover of *Rp*FNR. Difference in absorbance is shown at several timepoints over the 450–800 nm wavelength range. Concentrations of *Rp*FNR, 4.0 μM; NADPH, 50 μM; and duroquinone, 250 µM (after mixing). Spectra correspond to absorbance changes at 100 ms (1), 1 s (2), 5 s (3), and 20 s (4).

In order to characterize the reaction intermediates absorbing at 600 nm (Figure 6), the measurements were performed at different wavelengths. The results show that the absorbance initially increased in the range of 525–750 nm with λ_max_ ~ 720 nm (Figure 7). Subsequently, the formation and decay of a secondary flat absorbance band with a maximum at 600–700 nm took place (Figure 7). One may note that the formation of the transient species is not caused by the interaction of isoalloxazine ring of FAD and quinone, because the analogous transient absorbance spectra were obtained during the reoxidation of *Rp*FNR with oxygen (data not shown).

## 4. Discussion

Due to the large differences in the structure between plant-type and TrxR-type FNRs, the main focus of this work was to disclose the possible differences and similarities in their redox properties. In addition to the mechanistic aspects of studies of the reactions of *Rp*FNR with redox active xenobiotics, an important aspect is that *R. palustris* is a model microorganism of anaerobic metabolism of organic compounds [18] in which *Rp*FNR may be involved.

In this work, the redox potentials of a representative of TrxR-type FNR were identified for the first time. The *E*^0^_7_ of *Rp*FNR, −0.276 V (Figure 2A), is close to the redox potential of plant-type FNR from *P. falciparum,* −0.280 V [32], and less negative than that of spinach FNR, −0.342 V [8]. It is currently difficult to draw conclusions about the reasons for these differences. However, we can mention some factors that may lead to a relatively high FADH· stability, 26.5% at equilibrium, which is comparable to that of semiquinone of spinach FNR, 27% at pH 7.0 [8], and *Anabaena* FNR, 22% at pH 8.0 [33]. *Rp*FNR lacks the specific *Anabaena* FNR ion pair Ser80-Glu301 [34] (Ser96-Glu312 in spinach FNR [35]), which forms a H-bond with isoalloxazine N5 and most significantly contributes to FADH· stability. However, based on structural data of other FNRs and flavodoxins, the stability of FADH· may be enhanced by the π–π interaction of isoalloxazine with Tyr328 (Tyr98 in *Desulfovibrio vulgaris* flavodoxin [36], Tyr303 in *Anabaena* PCC7119 FNR [37]), formation of H-bonds of isoalloxazine N3 with the carboxy oxygen of Asp56 (Glu59 of *Clostridium beijerinckii* flavodoxin [38]), and O2 with the amide nitrogen of Ile300 (Ile356 of adrenodoxin reductase [39]). The data obtained suggest that the likely direction of electron transfer catalyzed by *Rp*FNR is the reduction of Fd at the expense of NADPH.

The “ping-pong” mechanism of quinone reduction by *Rp*FNR (Figure 3), pointing to the occurrence of separate reductive and oxidative half-reactions, is common to other FNRs [19,31]. As in our previous studies of *Anabaena* and *P. falciparum* FNR [19,31], no strict oxidant specificity in the reduction of quinones, aromatic nitrocompounds, and *N-*oxides by *Rp*FNR can be discerned from our data except for an increase in their log *k*_cat_/*K*_m_ with *E*^1^_7_ (Figure 4). This suggests a possible applicability of the “outer sphere” electron transfer model [40]. According to this model, the bimolecular rate constant of the electron transfer between the reactants (*k*_12_) is expressed as

*k*_12_ = (*k*_11_ × *k*_22_ × *K* × *f*)^1/2^, (7)

where *k*_11_ and *k*_22_ are the electron self-exchange rate constants of the reactants, *K* is the equilibrium constant of the reaction (log *K* = Δ*E*^1^/0.059 V), and *f* is expressed as

log *f* = (log *K*)^2^/4log (*k*_11_ × *k*_22_/*Z*^2^), (8)

where *Z* is the frequency factor, 10^11^ M^−1^s^−1^ [40]. According to Equations (7) and (8), in the reaction of the electron donor with a series of homologous oxidants (which display the same *k*_22_), log *k*_12_ will exhibit a parabolic (square) dependence on Δ*E*^1^ with a slope 8.45 V^−1^ at Δ*E*^1^ = ± 0.15 V. Because *k*_22_ = ~10^6^ M^−1^s^−1^, characteristic of nitroaromatics, is 100-fold lower than that of quinones and aromatic *N*-oxides, *k*_22_ = ~10^8^ M^−1^s^−1^, the reactivity of ArNO_2_ is about 10-fold lower when compared to quinones and ArN→O of similar *E*^1^_7_ values [41,42]. In this context, it can be noted that the reactivity of *Rp*FNR in reactions with both quinones and ArNO_2_, i.e., their *k*_cat_/*K*_m_, is close to that previously observed in reactions with plant-type *Pf*FNR and *Anabaena* FNR [19,31]. On the other hand, for reasons not yet known, AcPyP^+^ is 10 times better at oxidizing *Rp*FNR (Figure 2A) than *Pf*FNR [19]. Since the *k*_cat_ of reactions vary considerably (Table 1), it can be suggested that the limiting stage of the catalytic cycle is the oxidative half-reaction. The data of the Figure 5A show that the dominant mechanism of inhibition of the reaction product NADP^+^ is its competition with NADPH for binding to the oxidized form of the enzyme. The noncompetitive NADP^+^ inhibition with respect to the oxidant (Figure 5B) is slightly different from the uncompetitive inhibition of analogous *Anabaena* and *P. falciparum* FNR-catalyzed reactions [19,31]. This is most likely related to the binding of NADP^+^ to the reduced form of the enzyme with low affinity [16].

According to previous studies, photoreduced *Anabaena* FNR was reoxidized by quinones in two steps, FADH^−^ → FADH·, and FADH· → FAD, with the rate-limiting FADH· oxidation step [31,43]. This was evidenced by a transient formation of FADH· with 600 nm absorption. The reoxidation of *Rp*FNR should also involve single-electron transfer steps, since it reduces quinones in a predominantly single-electron way. Because the decay of the transient 600 nm absorption and the enzyme reoxidation monitored at 460 nm proceeds with a similar rate (Figure 6B), the FADH oxidation can be a rate-limiting step in quinone reduction by *Rp*FNR. However, the absorption characteristics of *Rp*FNR multiple turnover intermediates (Figure 7) differ from those of FADH· formed in the absence of NADP^+^ (Figure 2B), although over time they become more similar. Absorption above 700 nm is not characteristic of FADH [24] and indicates the parallel formation of other reaction intermediates. For example, FADH^−^–NADP^+^ charge-transfer complexes absorb up to 1000 nm [44]. However, this possibility is ruled out because the spectrum of the intermediates ends at 770–800 nm (Figure 7). In addition, the FADH^−^–NADP^+^ complexes possess ε ~1.0 mM^−1^cm^−1^ at 610–725 nm [45]; thus, they would give about three times less increase in absorption than we see in Figure 6. Most likely, the data in Figure 7 reflect the formation of FADH·–NADP(H) complexes observed in adrenodoxin reductase, which absorb at λ > 700 nm but are only partly characterized [46,47].

The catalytic cycle of *Rp*FNR should be characterized by significant movement of the FAD- and NADP(H)-binding domains relative to each other, including the motions of flexible C-terminal region [10,13,16,48]. Hypothetically, this could lead to shielding and deshielding of the FAD isoalloxazine ring, potentially complicating the access of oxidants. The electron exchange rate constants (*k*_11_) of metalloproteins can be used to estimate the electron transfer distance (*R*_p_) during the reactions with inorganic complexes at infinite ionic strength, where the electrostatic interactions are absent [49]

*R*_p_ (Å) = 6.3 − 0.35 ln *k*_11_. (9)

We applied this approach to the analysis of reactions of FNRs and other single-electron transferring flavoenzymes with uncharged aromatic oxidants, quinones, and nitroaromatics and obtained *R*_p_ levels of 5.0 Å (Q) and 4.4 Å (ArNO_2_) for *Anabaena* FNR, and 4.8–5.0 Å (Q) and 4.9–5.6 Å (ArNO_2_) for *P. falciparum* FNR [19]. However, a systematic overestimation of the electron transfer distances in this case is possible, because the dimethylbenzene part of the FAD isoalloxazine ring of the above enzymes is partly exposed to the solvent [2,5,16]. Therefore, the obtained values may be of limited usefulness only in approximately assessing the “intrinsic” flavoenzyme reactivity. For the reactions of *Rp*FNR with Q and ArNO_2_, the approximate *k*_11_ values may be obtained from the data of Figure 3 at Δ*E*^1^_7_ = 0, where *k*_12_ = (*k*_11_ × *k*_22_)^1/2^. At *E*^1^_7_ of the oxidant being equal to −0.285 V, the log *k*_11_ values are equal to 1.36 ± 0.46 (quinones) and 1.04 ± 0.22 (nitroaromatics), which then gives *R*_p_ = 5.2 ± 0.4 Å and *R*_p_ = 5.4 ± 0.2 Å, respectively, according to Equation (9). It can be noted that the possible uncertainty of the E-FADH· potential in the range of 10–15 mV has almost no effect on the *R*_p_ value, changing it by only 0.1 Å. Thus, these *R*_p_s are close to the *R*_p_ values for plant-type FNRs given above, indicating that possible steric interferences in the structure of *Rp*FNR may not affect the low *M*_r_ oxidant reduction rate. These data will be valuable in our further studies focusing on the specific features of the interaction of *Rp*FNR with its ferredoxin-type redox partners.

## 5. Conclusions

Despite structural differences, many of the redox properties of TrxR-type *Rp*FNR redox are similar to those of plant-type FNR: (i) the standard redox potential of FAD and its neutral semiquinone stability, (ii) single-electron reduction of quinones and nitroaromatic compounds, their reactivity and its dependence on single-electron reduction potential, (iii) transient FAD semiquinone formation, and (iv) calculated electron transfer distances. The slight differences in the action of plant-type and *Rp*FNR are manifested through the much faster reduction of AcPyP^+^ by *Rp*FNR and the different mode of its inhibition by the reaction product NADP^+^. These data may be useful in further studies of the specific interaction of *Rp*FNR with its ferredoxin-type redox partners.

## Figures and Tables

**Table 1:** Steady-state rate constants of the reduction of nonphysiological electron acceptors by 100 µM NADPH catalyzed by *Rp*FNR. The *E*^1^_7_ values of compounds taken from [22,28,29].

No.	Compound	*E*^1^_7_ (V)	*k_cat_*_(app)_ (s^−^^1^)	*k*_cat_/*K*_m_ (M^−^^1^s^−1^)
Quinones				
1	1,4-Benzoquinone	0.090	130 ± 16	9.4 ± 0.8 × 10^5^
2	2-CH_3_-1,4-benzoquinone	0.010	130 ± 12	5.6 ± 0.6 × 10^5^
3	2,6-(CH_3_)_2_-1,4-benzoquinone	−0.070	52.1 ± 1.8	2.1 ± 0.13 × 10^5^
4	5-OH-1,4-naphthoquinone	−0.090	138.5 ± 9.3	1.5 ± 0.23 × 10^6^
5	5,8-(OH)_2_-1,4-naphthoquinone	−0.110	45.4 ± 3.4	3.5 ± 0.2 × 10^6^
6	9,10-Phenanthrene quinone	−0.120	34.6 ± 2.4	2.0 ± 0.4 × 10^6^
7	1,4-Naphthoquinone	−0.150	110 ± 13	2.3 ± 0.4 × 10^5^
8	2-CH_3_-1,4-naphthoquinone	−0.200	21.6 ± 2.1	6.0 ± 0.8 × 10^4^
9	(CH_3_)_4_-1,4-benzoquinone(duroquinone)	−0.260	4.07 ± 0.53	1.1 ± 0.1 × 10^4^
10	9,10-Anthraquinone-2-sulphonate	−0.380	3.56 ± 0.33	1.0 ± 0.16 × 10^4^
11	2-OH-1,4-naphthoquinone	−0.410	0.26 ± 0.03	3.1 ± 0.2 × 10^3^
12	2-CH_3_-3-OH-1,4-naphthoquinone	−0.460	1.35 ± 0.13	4.8 ± 0.4 × 10^3^
Nitroaromatic compounds				
13	Tetryl	−0.191	5.69 ± 0.14	4.35 ± 0.30 × 10^4^
14	*N*-methylpicramide	−0.225	1.93 ± 0.26	4.8 ± 0.6 × 10^3^
15	2,4,6-Trinitrotoluene	−0.253	1.30 ± 0.13	2.43 ± 0.14 × 10^3^
16	Nifuroxime	−0.255	4.40 ± 0.32	6.9 ± 0.4 × 10^3^
17	Nitrofurantoin	−0.255	2.21 ± 0.12	5.1 ± 0.5 × 10^3^
18	*p*-Dinitrobenzene	−0.257	2.21 ± 0.35	3.1 ± 0.2 × 10^3^
19	*o*-Dinitrobenzene	−0.287	0.48 ± 0.07	1.28 ± 0.2 × 10^3^
20	4-Nitrobenzaldehyde	−0.325	0.97 ± 0.13	2.38 ± 0.4 × 10^3^
21	3,5-Dinitrobenzoic acid	−0.344	0.09 ± 0.01	2.91 ± 0.2 × 10^3^
22	*m*-Dinitrobenzene	−0.348	0.42 ± 0.06	9.6 ± 0.7 × 10^2^
23	4-Nitroacetophenone	−0.355	0.30 ± 0.05	8.0 ± 0.67 × 10^2^
24	CB-1954	−0.385	0.52 ± 0.05	1.75 ± 0.14 × 10^3^
25	4-Nitrobenzyl alcohol	−0.475	0.23 ± 0.03	2.50 ± 0.16 × 10^2^
Aromatic *N*-oxides				
26	7-F-tirapazamine	−0.400	1.20 ± 0.11	1.80 ± 0.31 × 10^3^
27	Tirapazamine	−0.456	0.53 ± 0.04	9.41 ± 0.82 × 10^2^
28	7-C_2_H_5_O-tirapazamine	−0.494	0.46 ± 0.03	4.91 ± 0.32 × 10^3^
Single-electron acceptors				
29	Ferricyanide ^a^	0.410	394 ± 19	8.8 ± 1.0 × 10^6^
30	Fe(EDTA)^−^	0.120	1.2 ± 0.1	2.4 ± 0.2 × 10^3^
31	Benzylviologen	−0.354	19.6 ± 2.3	3.6 ± 0.3 × 10^4^

^a^ On the single-electron basis.

## Data Availability

Data is contained within the article.
